# Supplementary material for: Sensory Entrained TMS (seTMS) Enhances Motor Cortex Excitability
Source: Hum Brain Mapp. 2025 Jul 7;46(10):e70267. doi: 10.1002/hbm.70267 (PMC12231655; doi:10.1002/hbm.70267)
Supplement: Supplementary file 1 — Data S1 Supporting Information. [file HBM-46-e70267-s001.pdf]

Supplementary

Table S1. Demographics. *n*=27

Age, mean years (SD)	39.6 (14.2)
Sex	
Female, *n* (%)	14 (51.9)
Male, *n* (%)	12 (44.4)
Other or prefer not to state, *n* (%)	1 (3.7)
Handedness	
Left hand dominant, *n* (%)	2 (7.4)
Right hand dominant, *n* (%)	25 (92.6)
Ambidextrous, *n* (%)	0 (0.0)
Education	
GED or High School Diploma, *n* (%)	1 (3.7)
Some college, no degree, *n* (%)	2 (7.4)
Two year degree, *n* (%)	4 (14.8)
Four year degree, *n* (%)	13 (48.2)
Post graduate degree, *n* (%)	7 (25.9)
Employment	
Part-time, *n* (%)	9 (33.3)
Full-time, *n* (%)	7 (25.9)
Unemployed, *n* (%)	8 (29.6)
Retired, *n* (%)	1 (3.7)
Part-time student, *n* (%)	0 (0.0)
Full-time student, *n* (%)	2 (7.4)
Race	
White, *n* (%)	12 (44.4)
Black or African American, *n* (%)	4 (14.8)
American Indian or Alaska Native, *n* (%)	0 (0.0)
Asian, *n* (%)	9 (33.3)
Native Hawaiian or Other Pacific Islander, *n* (%)	1 (3.7)
Two or more races, *n* (%)	0 (0.0)
Some other race or prefer not to state, *n* (%)	1 (3.7)

Table S2. Demographics. *n*=19

Age, mean years (SD)	37.7 (14.3)
Sex	
Female, *n* (%)	8 (42.1)
Male, *n* (%)	10 (52.6)
Other or prefer not to state, *n* (%)	1 (5.3)
Handedness	
Left hand dominant, *n* (%)	1 (5.3)
Right hand dominant, *n* (%)	18 (94.7)
Ambidextrous, *n* (%)	0 (0.0)
Education	
GED or High School Diploma, *n* (%)	0 (0.0)
Some college, no degree, *n* (%)	2 (10.5)
Two year degree, *n* (%)	2 (10.5)
Four year degree, *n* (%)	10 (42.1)
Post graduate degree, *n* (%)	5 (10.5)
Employment	
Part-time, *n* (%)	4 (21.0)
Full-time, *n* (%)	8 (42.1)
Unemployed, *n* (%)	4 (21.0)
Retired, *n* (%)	1 (5.3)
Part-time student, *n* (%)	1 (5.3)
Full-time student, *n* (%)	1 (5.3)
Race	
White, *n* (%)	8 (42.1)
Black or African American, *n* (%)	2 (10.5)
American Indian or Alaska Native, *n* (%)	0 (0.0)
Asian, *n* (%)	7 (36.8)
Native Hawaiian or Other Pacific Islander, *n* (%)	1 (5.3)
Two or more races, *n* (%)	0 (0.0)
Some other race or prefer not to state, *n* (%)	1 (5.3)

Table S3. Demographics. *n*=13

Age, mean years (SD)	36.1 (12.0)
Sex	
Female, *n* (%)	5 (38.5)
Male, *n* (%)	7 (53.8)
Other or prefer not to state, *n* (%)	1 (7.7)
Handedness	
Left hand dominant, *n* (%)	1 (7.7)
Right hand dominant, *n* (%)	12 (92.3)
Ambidextrous, *n* (%)	0 (0.0)
Education	
GED or High School Diploma, *n* (%)	0 (0.0)
Some college, no degree, *n* (%)	2 (15.4)
Two year degree, *n* (%)	2 (15.4)
Four year degree, *n* (%)	7 (53.8)
Post graduate degree, *n* (%)	2 (15.4)
Employment	
Part-time, *n* (%)	4 (30.8)
Full-time, *n* (%)	6 (46.2)
Unemployed, *n* (%)	3 (23.1)
Retired, *n* (%)	0 (0.0)
Part-time student, *n* (%)	0 (0.0)
Full-time student, *n* (%)	0 (0.0)
Race	
White, *n* (%)	5 (38.5)
Black or African American, *n* (%)	2 (15.4)
American Indian or Alaska Native, *n* (%)	0 (0.0)
Asian, *n* (%)	5 (38.5)
Native Hawaiian or Other Pacific Islander, *n* (%)	1 (7.7)
Two or more races, *n* (%)	0 (0.0)
Some other race or prefer not to state, *n* (%)	0 (0.0)

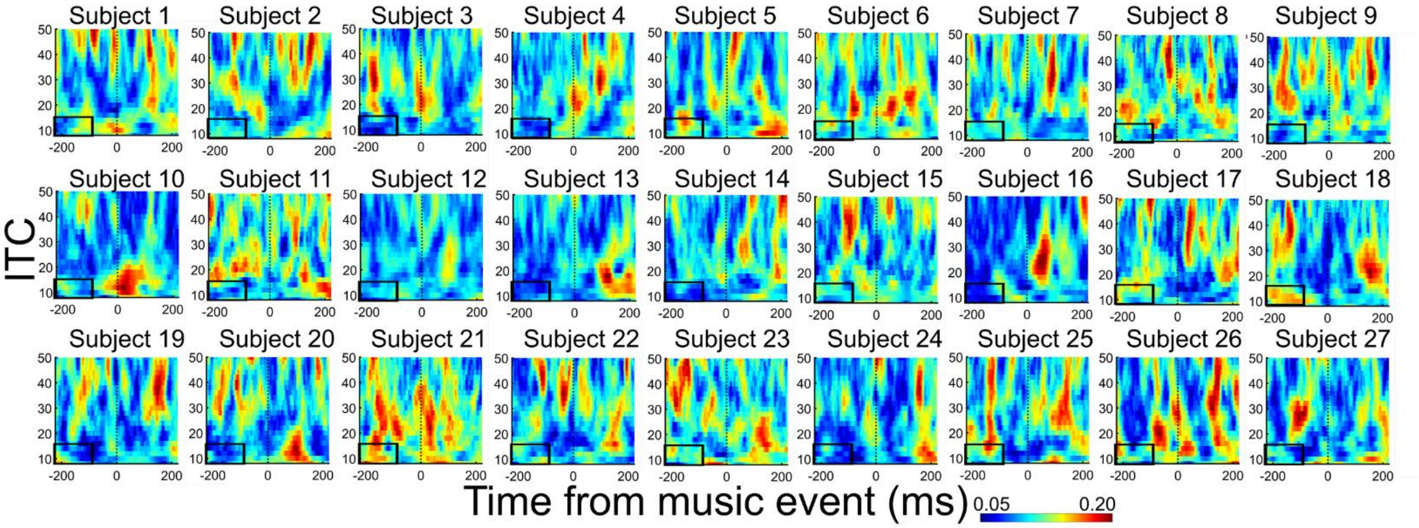

Fig. S1. Individual participant ITC, averaged across three channels from over the motor cortex (C5, C3, C1) and with frequency/time window ROI indicated with a black box (*n*=27)**.**

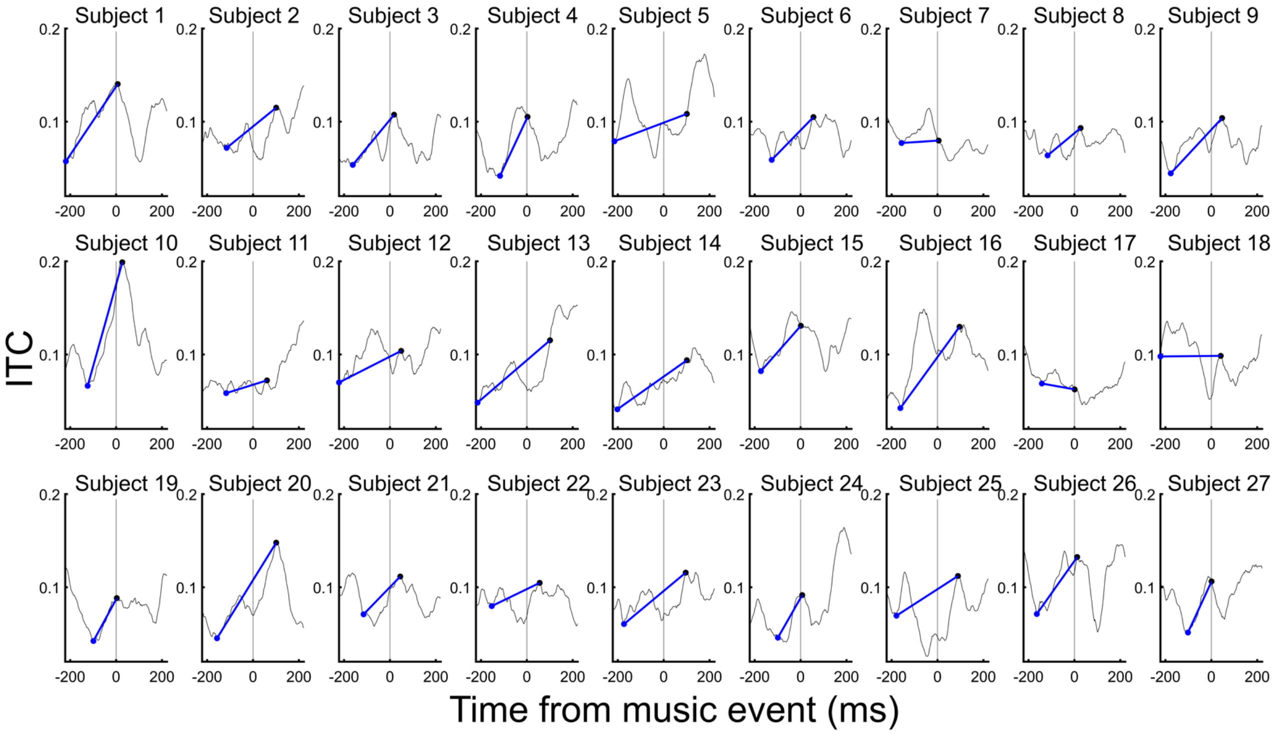

Fig. S2. Individual participant ITC dynamics. Shown are alpha ITC with local minima and maxima and slope (*n*=27).

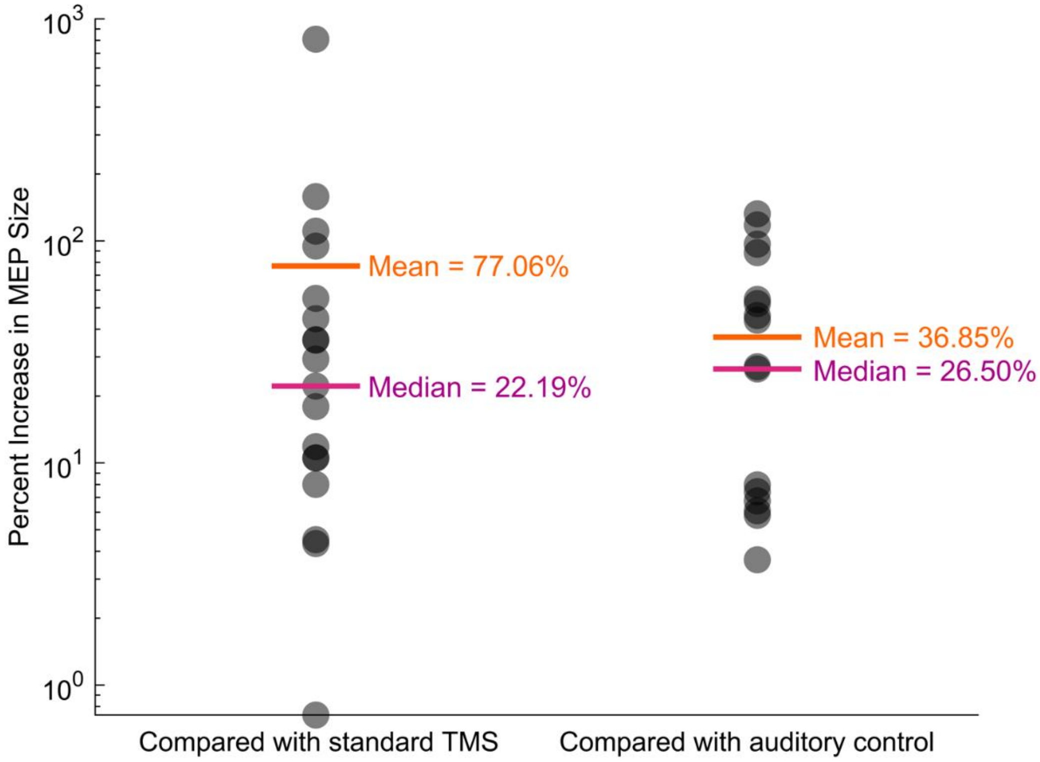

Fig. S3. Percent increase in MEP size with seTMS, compared to standard TMS (left) and to the auditory control (right). Mean and median percent increase is shown in orange and magenta, respectively (*n*=19).

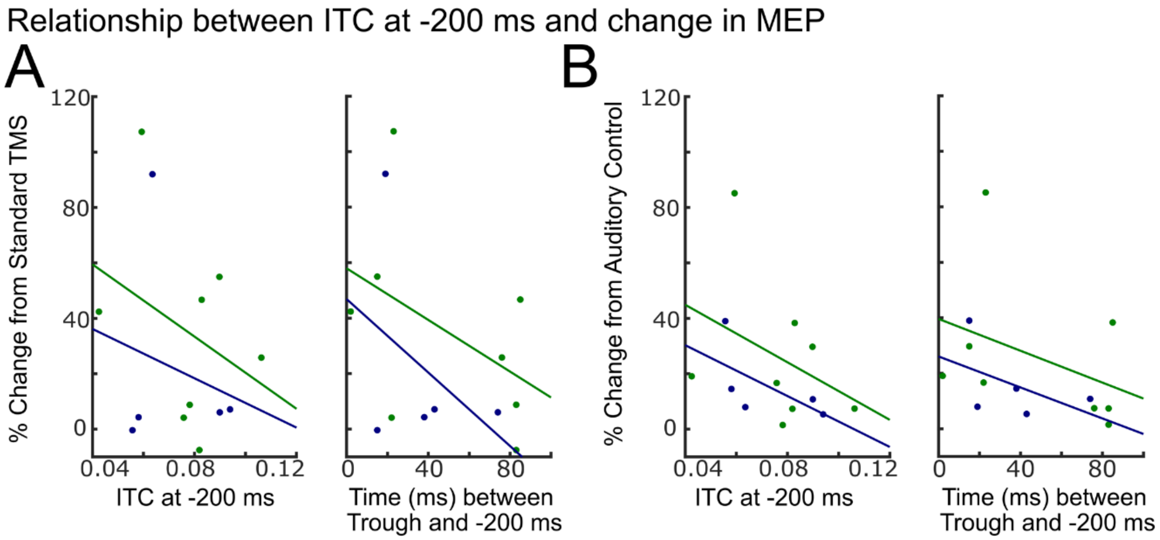

Fig. S4. Relationship between ITC and increase in MEP size, in musicians and nonmusicians (*n*=13). Results shown are using ITC at -200 ms and using individual participant ITC trough time, and increase in MEP size was calculated using the A) standard TMS and B) auditory control conditions.

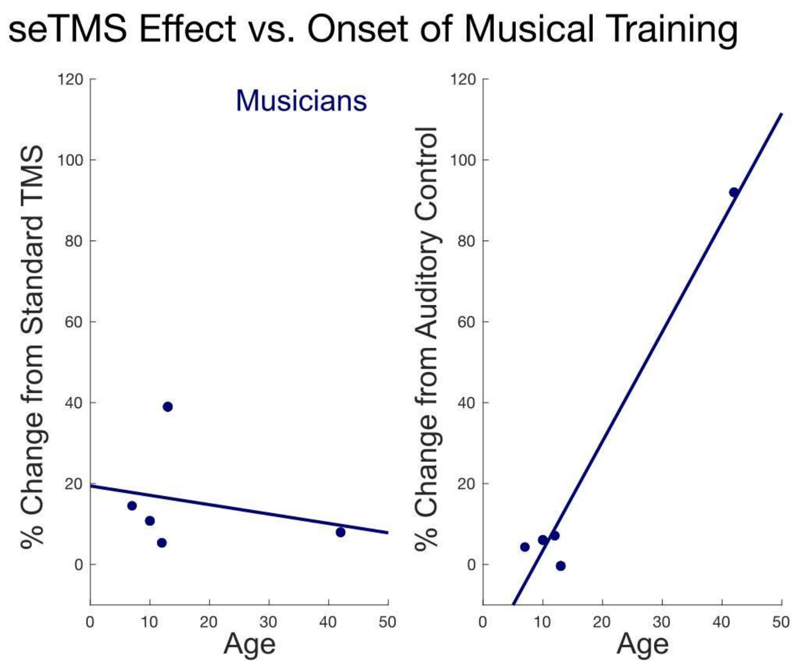

Fig. S5. seTMS effect by age at which musical training began amongst musicians (*n*=5).

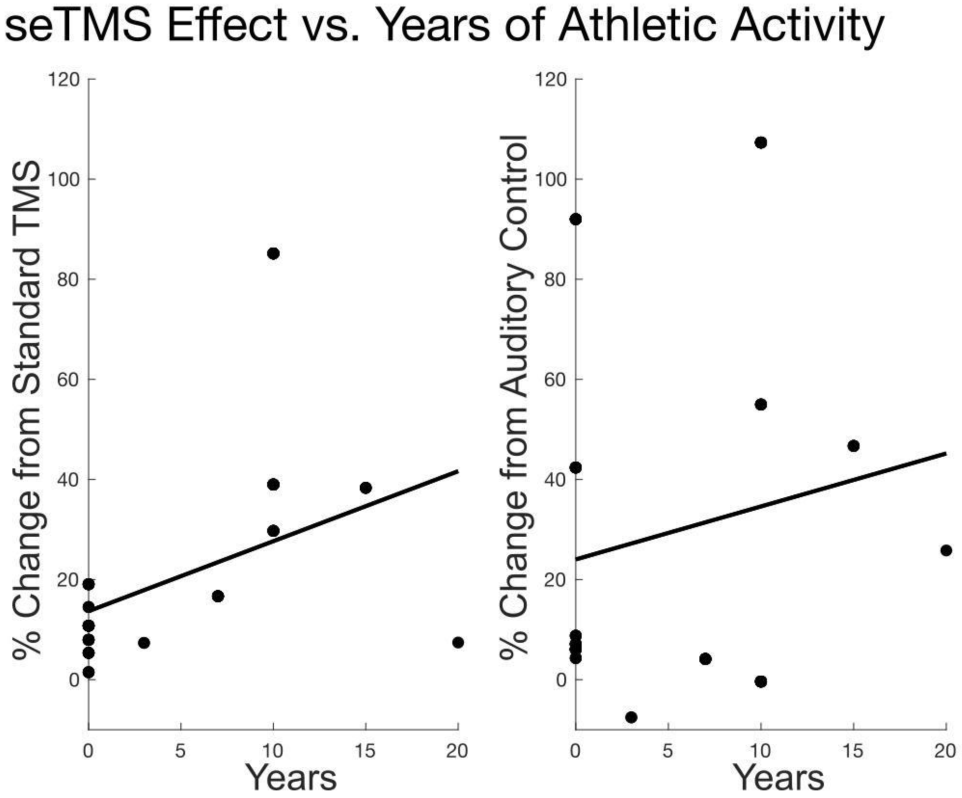

Fig. S6. seTMS effect for all subjects by years of athletic activity (*n*=13).

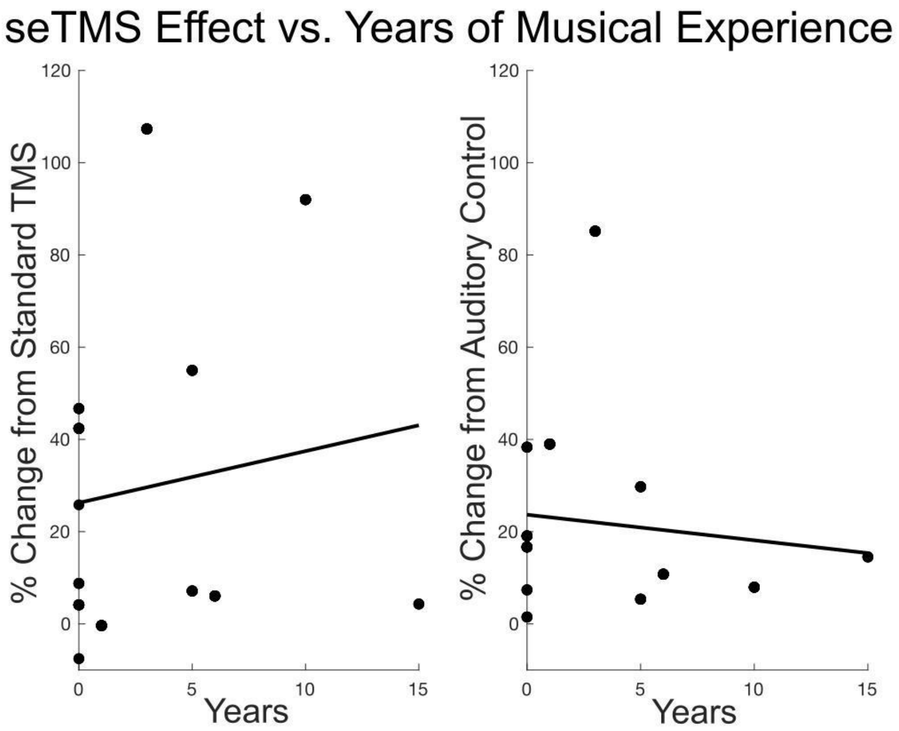

Fig. S7. seTMS effect for all subjects by years of musical training or experience (*n*=13).

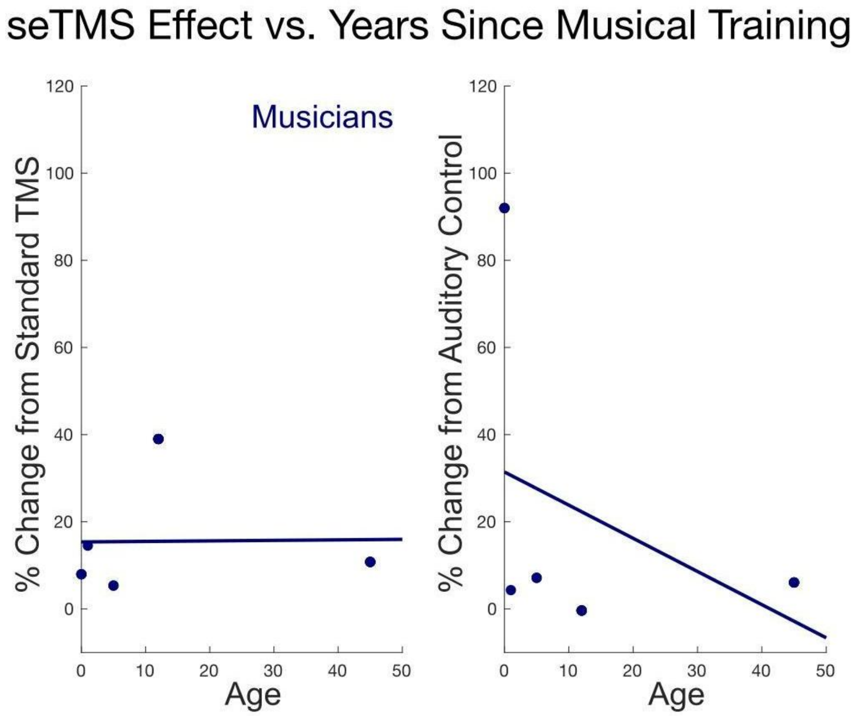

Fig. S8. seTMS effect by years since most recent musical training or experience amongst musicians (*n*=5).

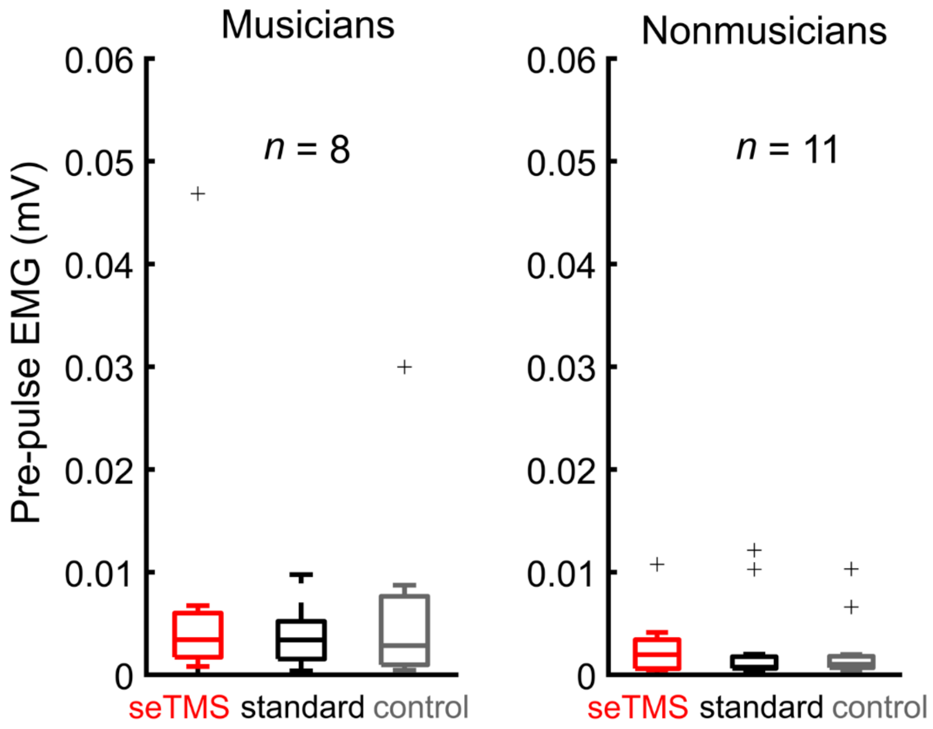

58 **Fig. S9. Pre-pulse EMG.** Calculated as the absolute value of EMG between 20-5 ms prior to the
59 TMS pulse.

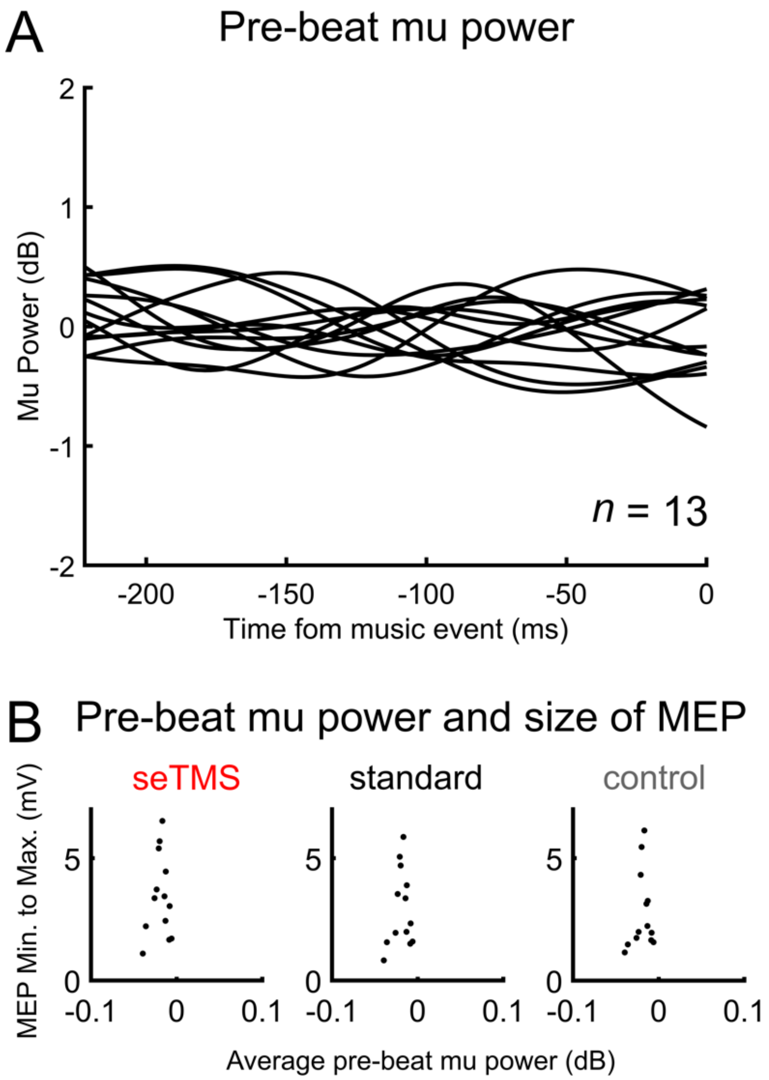

Fig. S10. Pre-beat mu power (*n*=13). A) Power averaged over the channels in the ROI, shown across the pre-beat period. B) Pre-beat mu power averaged over the pre-beat period by MEP size from each of the conditions.
